# Mechanosignaling activation of TGFβ maintains intervertebral disc homeostasis

**DOI:** 10.1038/boneres.2017.8

**Published:** 2017-03-21

**Authors:** Qin Bian, Lei Ma, Amit Jain, Janet L Crane, Khaled Kebaish, Mei Wan, Zhengdong Zhang, X Edward Guo, Paul D Sponseller, Cheryle A Séguin, Lee H Riley, Yongjun Wang, Xu Cao

**Affiliations:** 1 Department of Orthopaedic Surgery, Institute for Cell Engineering, Johns Hopkins University, Baltimore, MD, USA; 2 Institute of Spine, Longhua Hospital, Shanghai University of Traditional Chinese Medicine, Shanghai, PR China; 3 Department of Pediatrics, Johns Hopkins University, Baltimore, MD, USA; 4 Department of Biomedical Engineering, Columbia University, NY, USA; 5 Departments of Physiology and Pharmacology, University of Western Ontario, London, Canada

## Abstract

Intervertebral disc (IVD) degeneration is the leading cause of disability with no disease-modifying treatment. IVD degeneration is associated with instable mechanical loading in the spine, but little is known about how mechanical stress regulates nucleus notochordal (NC) cells to maintain IVD homeostasis. Here we report that mechanical stress can result in excessive integrin α_v_β_6_-mediated activation of transforming growth factor beta (TGFβ), decreased NC cell vacuoles, and increased matrix proteoglycan production, and results in degenerative disc disease (DDD). Knockout of TGFβ type II receptor (TβRII) or integrin α_v_ in the NC cells inhibited functional activity of postnatal NC cells and also resulted in DDD under mechanical loading. Administration of RGD peptide, TGFβ, and α_v_β_6_-neutralizing antibodies attenuated IVD degeneration. Thus, integrin-mediated activation of TGFβ plays a critical role in mechanical signaling transduction to regulate IVD cell function and homeostasis. Manipulation of this signaling pathway may be a potential therapeutic target to modify DDD.

## Introduction

Degenerative disc disease (DDD) remains a common musculoskeletal disorder that brings an enormous socioeconomic burden.^1–3^ Although numerous factors associated with DDD have been identified, the exact molecular pathogenesis of DDD has yet to be elucidated. The current treatments focus on symptomatic relief from pain through injections, physical therapy, and activity modification^4^ or surgical intervention such as disc decompression, spinal fusion, and disc replacement.^3,5^ However, none of these interventions halt the progression of degeneration nor restore the physiologic disc function.

Dysfunction of nucleus pulposus (NP) cells is the key in the onset of intervertebral disc (IVD) degeneration.^1,6–8^ It is known that NP cells are of notochord origin,^9–11^ termed as notochordal (NC) cells at early age. NC cells are large with intracellular vacuoles making up at least 25% of the cell area.^7,8^ The large vacuoles generate IVD space during spinal morphogenesis.^9,12–14^ During maturation and degeneration, the NC cells undergo morphologic and functional transition with the loss of their vacuoles. The resultant fibroblast-like cells have decreased the expression of extracellular matrix protein such as aggrecan,^15^ which enables the NP to maintain height and turgor against compressive loads via its osmotic properties.^16,17^ The mechanism driving NC cell transition is unclear, particularly how the mechanical load influences cell signaling.

Temporal–spatial activation of latent matrix transforming growth factor beta (TGFβ) has been shown to modulate chondrocyte anabolic activity in articular cartilage, maintain bone homeostasis during bone remodeling, and help with tissue repair.^18,19^ The α_v_ integrins in combination with -β_6_, -β_5_, and -β_8_ have been shown to mediate the activation of TGFβ.^20–24^ Integrins enable cells to transduce mechanical loads into biological signaling. As NP cells express α_v_ and multiple β integrin subunits, integrin-mediated activation of TGFβ may play a critical role in IVDs.^25^ In addition, active TGFβ is known to act upstream of connective tissue growth factor (CTGF/CCN2) and aggrecan, both of which are involved in DDD development.^26,27^ Thus, we sought to understand the role of TGFβ in IVD homeostasis.

In this study, we systematically investigated the role of mechanical stress on the functional transition of NC cells and IVD homeostasis. Utilizing multiple rodent models, we found that mechanical stress resulted in integrin α_v_β_6_-mediated activation of TGFβ. Abnormal stress resulted in excessive TGFβ signaling and accelerated NC cells functional transition. Administration of RGD peptide and neutralizing antibodies against TGFβ and α_v_β_6_ attenuated these changes. On the other hand, conditional knockout of TβRII or α_v_ also impeded NC cells’ transition and caused IVD degeneration by mechanical stimuli. Thus, precise integrin-induced activation of TGFβ is required to maintain IVD cell function and homeostasis.

## Materials and methods

### Subjects

#### Animal models

##### Lumbar spine instability mouse model

C57BL/6J (male, 8-week old) mice were purchased from Charles River, Wilmington, MA, USA. After anesthetized with ketamine and xylazine, they were operated by resection of the lumbar 3th–lumbar 5th (L_3_–L_5_) spinous processes along with the supraspinous and interspinous ligaments to induce instability of lumbar spine.^28,29^ Sham operations were carried out only by detachment of the posterior paravertebral muscles from the L_3_–L_5_ vertebrae. The operated mice were intraperitoneally injected with either TβRI inhibitor (SB-505124, Sigma-Aldrich, St Louis, MO, USA) at a dose of 1 mg·kg^−1^ (SB group) or the equivalent volume of vehicle (dimethyl sulfoxide; Veh group) once every 2 days. Mice (8-week old) were killed at 0, 1, 2, 4, and 8 weeks after the surgery (*n*=6 per group).

##### Caudal spine instability mouse model

The instability of caudal spine was induced by fully depth annular stab and NP removal of the caudal 7th–8th (C_7–8_) IVD.^30^ The adjacent C_8–9_ IVDs were chosen for observation at 4 weeks post surgery (*n*=6 per group). The treatments were the same as those described in the first model.

##### Rat caudal IVD compression model

Twelve-week-old male Sprague–sDawley rats (Charles River) were attached at caudal vertebrae with a loading device.^31–34^ In detail, two 0.7366 mm stainless steel Kirchner wires were inserted percutaneously into each of the C_8_ and C_10_, and attached to 35 mm external diameter aluminum rings. Rings were pre-coated with a resin and connected longitudinally with four stainless steel threaded rods. Axial loads were applied by four calibrated springs (0.50 N·mm^−1^) installed over each rod. In the vehicle (Veh) and 1D11 group, the axial stress loaded from the distal side produced a calculated compressive pressure of 1.3 MPa onto C_8–9_ and C_9–10_ IVDs through tightening calibrated springs.^31,33,34^ The C_8–9_ of loaded IVDs was injected with an alginate bead (1 μL) containing 0.7 μg 1D11 (TGFβ-neutralizing antibody; R&D Systems, Minneapolis, MN, USA),^18^ whereas the C_9–10_ IVDs with that containing vehicle (*n*=6) once during the surgery. In the sham group, the rats were also attached with the loading device at C_8_ and C_10_, but no compressive pressure was exerted onto the IVDs. Both C_8–9_ and C_9–10_ were injected with vehicle-containing alginate bead. The rats were killed at 2 weeks after the surgery (*n*=6 per group).

*Noto-cre* (CD1 background mouse expressing Cre recombinase driven by *Noto* promoter) mouse strain was obtained from the lab of Cheryle A. Séguin.^10^ *TgfβRII*^*flox/flox*^ mouse strain (C57BL/6J background mouse homozygous for *TgfβRII* flox allele) was obtained from the lab of H.L. Moses.^35^ *Noto*^*cre*^::*TgfβRII*^*flox/+*^ mice were generated by crossing heterozygote *Noto-cre* strain with *homozygote TgfβRII*^*flox/flox*^ strain. *TgfβRII*^*−/−*^ and *TgfβRI*^*+/+*^ (wild-type (WT) littermates) offspring were generated by crossing two *Noto*^*cre*^::*TgfβRII*^*flox/+*^ strains (*Noto*^*cre*^::*TgfβRII*^*flox/flox*^ mice that are *TgfβRII* conditional deletion in *Noto* lineage cells are referred to as “*TgfβRII*^*−/−*^”. *Noto*^*cre*^::*TgfβRII*^*+/+*^ mice that are heterozygous for *Noto-cre* are referred to as “*TgfβRII*^*+/+*^” in the text, *n*=6 per group).

The genotype of the mice was determined by PCR analyses of genomic DNA isolated from mouse tails using the following primers: *Noto*-directed *cre* forward, 5′- ATACCGGCAGATCATGCAAGC-3′, and reverse, 5′- ATGCACATATGCAACCCACA-3′.^10^ The *loxP TgfβRII* allele was identified with the primers forward, 5′- TAAACAAGGTCCGGAGCCCA-3′, and reverse, 5′- ACTTCTGCAAGAGGTCCCCT-3′.^36^

α_*v*_^*flox/flox*^ mice mouse strain was obtained from the lab of Andrew Burich, Benaroya Research Institute at Virginia Mason. *Noto*^*cre*^::α_*v*_^*flox/flox*^ mice that are α_*v*_ conditional deletion in *Noto* lineage cells are referred to as “α_*v*_^*−/−*^”. *Noto*^*cre*^:: α_*v*_^*+/+*^ mice that are heterozygous for *Noto-cre* are referred to as “α_*v*_^*+/+*^” in the text (*n*=3 per group). The *loxP* α_*v*_ allele was identified with the primers forward, 5′- TTCAGGACGGCACAAAGACCGTTG-3′, and reverse, 5′- CACAAATCAAGGATGACCAAACTGAG-3′.

C57BL/6J mice of the following ages: 16.0-day pregnant, 3 days, 1 weeks, 1 month, 2 months, 3 months and 4 months were purchased from Charles River (*n*=6 per time point).

All animals (rat and mouse) were maintained in the Animal Facility of the Johns Hopkins University School of Medicine. The experimental protocols for both species were reviewed and approved by the Institutional Animal Care and Use Committee of the Johns Hopkins University, Baltimore, MD, USA.

#### IVD *ex vivo* compression model

The L_1_–L_5_ lumbar IVDs were removed under sterile conditions from 4-week-old C57BL/6J mice. The collected IVDs were organ-cultured in Dulbecco’s modified Eagle’s medium (Invitrogen, Carlsbad, CA, USA) supplemented with 1% penicillin–streptomycin (MediaTech, Dallas, TX, USA) and pretreated with one of the followings: 2 ng·mL^−1^ of recombinant mouse TGFβ1 (7666-MB-005, R&D, Minneapolis, Minnesota, USA), 5.0 μg·ml^−1^ of 1D11, 2 μmol·L^−1^ of RGD peptide (3498, Tocris Bioscience, Burlington, NC, USA), 100 μg·mL^−1^ of anti-α_V_β_6_-neutralizing antibody (ab77906, Abcam, Cambridge, MA, USA) or Veh (13C4, R&D Systems) overnight. Compression loads of 0 or 1.0 MPa were applied by vertically placing weights on the IVDs after pretreatment. Loading duration was 24 h (*n*=6 per group).^37^

We determined the sample size based on power analysis via website http://www.biomath.info (for two groups, 80% power, 5% significance, two-sided), or via G*Power (for more than two groups, 80% power, 5% significance). For animal model and *TgfβRII* knockout studies, each experiment was conducted twice: one pilot experiment in three samples and two independent experiments of six samples. For α_*v*_ knockout mice, three independent experiments with six samples were conducted.

### Micro Computed Tomography (μCT)

The lower thoracic and whole lumbar spine from mice were dissected, fixed in 10% buffered formalin for 48 h, transferred into phosphate-buffered saline, and then examined by high-resolution μCT (Skyscan1172). The ribs on the lower thoracic were included for the identification of L_4_–L_5_ IVD localization. Images were reconstructed and analyzed using NRecon v1.6 and CTAn v1.9 (Skyscan company, San Jose, CA, USA), respectively. Three-dimensional model visualization software, CTVol v2.0 (Skyscan company, San Jose, CA, USA), was used to analyze parameters of the L_4_–L_5_ IVD with half height of L_4_ and L_5_ vertebrae. The scanner was set at a voltage of 49 kVp, a current of 200 μA, and a resolution of 6.8 μm per pixel to measure the IVD and endplate (EP). A resolution 16.8 μm of per pixel was set for the whole L_5_ vertebral body measurement. Coronal images of the L_4_–L_5_ IVD were used to perform three-dimensional histomorphometric analyses of IVD. IVD volume was defined by the region of interest to cover the whole invisible space between L_4_ and L_5_ vertebrae. Parameter: TV (total tissue volume) was used for three-dimensional structural analysis.

### Histochemistry, immunohistochemistry, and histomorphometry

The specimens were fixed in 10% buffered formalin for 48 h, decalcified in 10% EDTA (pH 7.4) for 14 days, dehydrated, and embedded in paraffin. Four-micrometer-thick coronal-oriented sections of the L_4_–L_5_, C_7–9_, or C_8–10_ spine were processed for safranin O and fast green staining. Tartrate-resistant acid phosphatase staining was performed using a standard protocol (Sigma-Aldrich). Immunostaining was performed using a standard protocol. Sections were incubated with primary antibodies to mouse aggrecan (1:200, AB1031, Millipore, Billerica, MA, USA), CCN2 (1:400, ab6992, Abcam), pSmad2/3 (1:200, sc-11769, Santa Cruz, Dallas, TX, USA), integrin α_V_β_6_ (1:100, bs-5791R-Biotin, Bioss, Woburn, MA, USA), integrin α_V_β_3_ (1:100, bs-1310R, Bioss), integrin α_V_β_5_ (1:100, bs-1356R, Bioss), integrin β_8_ (1:300, ab80673, Abcam), integrin α_V_ (Santa Cruz, 1:100, sc-6617-R), integrin β_6_ (Santa Cruz, 1:100, sc-6632), and TGFβRII (1:100, sc-400, Santa Cruz) at 4 °C overnight. For immunohistochemical staining, a horseradish peroxidase-streptavidin detection system (Dako, Carpinteria, CA, USA) was subsequently used to detect the immunoactivity, followed by counterstaining with hematoxylin (Sigma-Aldrich). For immunofluorescent assay, the slides were incubated with secondary antibodies conjugated with fluorescence at room temperature for 1 h while avoiding light. Morphometric study was performed by an image autoanalysis system (Olympus DP71, SEG Biological Microscopes, Center Valley, Pennsylvania, USA). Triplicates of each sample were used for staining.

### Quantitative histomorphometric analysis

Quantitative histomorphometric analysis was conducted in a blinded manner with Image-Pro Plus Software version 6.0 (Media Cybernetics Inc, Rockville, Maryland, USA). EP and IVD scores were obtained as previously described.^1,38^ The percentage of pSmad2/3-positive cells was obtained by counting the number of positive staining cells to the number of total cells in the NP region. The expression of integrin α_V_β_6_ was calculated by the sum of integrated optical density in the NP region. The area of CCN2-positive staining was calculated in the whole L_4_–L_5_ IVD in lumbar spine instability (LSI) mice (2-month old). In other experiments, the area of CCN2-positive staining was calculated only in the NP region. The percentage of Acan-positive staining was calculated by counting the positive staining area of region of interest that covers all cells in NP.

### Quantitative reverse transcription-PCR

Total RNA was extracted from NP tissue of IVD in *ex vivo* assay using TRIzol reagent (Sigma-Aldrich) according to the manufacturer’s instruction. The yield and purity of RNA were estimated spectrophotometrically using theA260/A280 ratio. Two micrograms of RNA was reverse-transcribed into complementary DNA using the SuperScript first-strand synthesis system (Invitrogen). One microliter of complementary DNA was subjected to quantitative reverse transcription-PCR amplification using SYBR GREENPCR Master Mix (Promega, Madison, WI, USA) and sequence-specific primers for Acan: 5′- CAGATGGCACCCTCCGATAC-3′and 5′- GACACACCTCGGAAGCAGAA-3′. The value of gene expression was normalized relative to the mouse GAPDH: 5′- AATGTGTCCGTCGTGGATCTGA-3′ and 5′- AGTGTAGCCCAAGATGCCCTTC-3′. PCR reactions were performed in triplicates. The data were analyzed using the 2^−ΔΔCT^ method.

### Western blot

Western blot analyses were conducted on the protein extraction from NP tissue in *ex vivo* assay. The protein extraction was centrifuged, and the concentration of supernatants was evaluated by DC protein assay (Bio-Rad Laboratories, Hercules, CA, USA), then the proteins were separated by SDS-polyacrylamide gel electrophoresis and blotted on a polyvinylidene fluoride membrane (Bio-Rad Laboratories). After incubation in specific antibodies, proteins were detected using an enhanced chemiluminescence kit (Amersham Biosciences, Pittsburgh, PA, USA). The target protein concentrations were examined by antibodies recognizing mouse pSmad2 (1:1 000, 3101, Cell Signaling Technology Inc., Danvers, MA, USA), Smad2 (1:1 000, 3103, Cell Signaling Technology Inc.), integrin α_V_ (1:500, sc-6617-R, Santa Cruz), and GAPDH (1:1 000, 8884, Cell Signaling Technology Inc.).

### Statistics

The data were expressed as mean±s.d., and statistical significance was determined using a Student’s *t*-test in time point or genetic mice comparison, or one-way analysis of variance followed by a *post hoc* Least-Significance-Difference (LSD) test (homogeneity of variance) or a Tukey’s test (heterogeneity of variance) in treatment or *ex vivo* assay comparison. The level of significance was defined as *P*<0.05. All data analyses were performed using SPSS 15.0 analysis software (SPSS Inc, Chicago, Illinois, USA).

## Results

### Activation of TGFβ associates with reduced NC cell vacuoles and increased extracellular proteoglycan in response to mechanical stress

**Figure 1:**
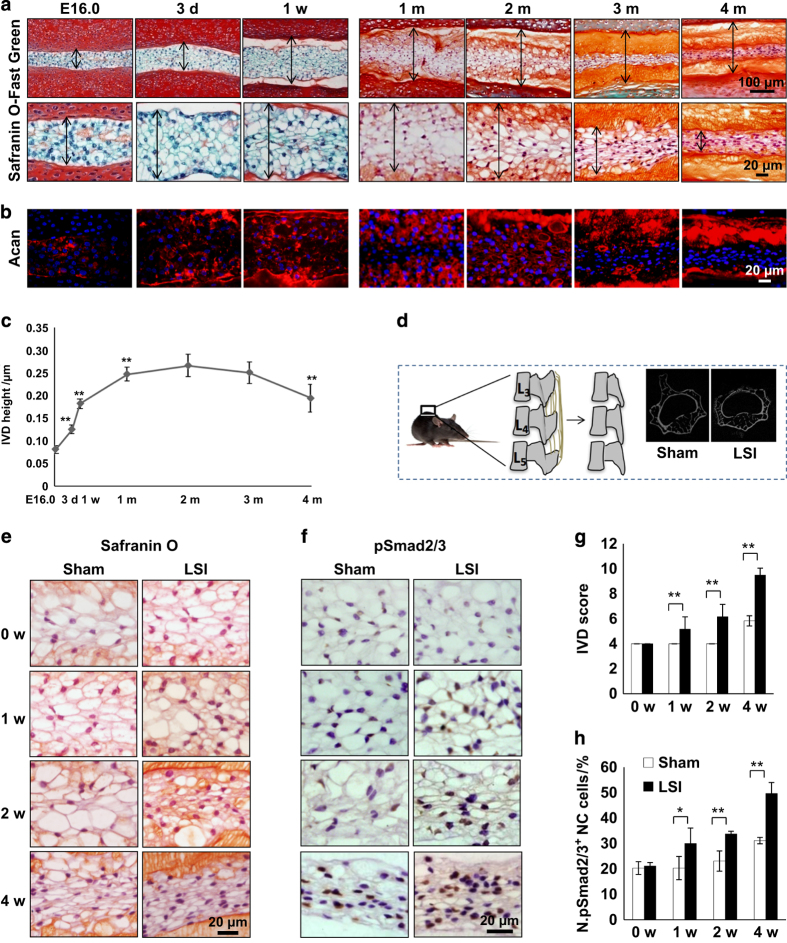
Mechanical instability induces activation of TGFβ, and reduces NC cell vacuoles. (**a**) Safranin O-Fast green staining of IVD sections from wild-type mice from E-16.0 to 4 months after birth. (**b**) Immunostaining of IVD sections showing the expression of aggrecan. *n=6* per time point. (**c**) The height of IVD indicated by the double-headed arrow was measured in **a**. (**d**) Lumbar spine instability mouse model (LSI). Mouse L_3_–L_5_ spinous processes were resected along with the supraspinous and interspinous ligaments to induce instability of lumbar spine. (This diagram was drawn by the author and has been published in *Scientific Reports*: http://www.nature.com/articles/srep27093/figure/1). (**e**) Representative Safranin O staining images of the IVD sections showing the changes of NC cells in LSI and sham-operated 2-month-old mice at 0, 1, 2 and 4 weeks (w) post surgery. (**f**) Representative immunostaining images of IVD sections with antibody against pSmad2/3 (brown). Hematoxylin stains nuclei purple. (**g**) Evaluation of IVD degeneration by IVD score. (**h**) Quantification of pSmad2/3^+^ cells in f. *n=6* per group. Data are shown as mean±s.d. *P<0.05, **P<0.01 (two-sided Student’s *t* test).

**Figure 2:**
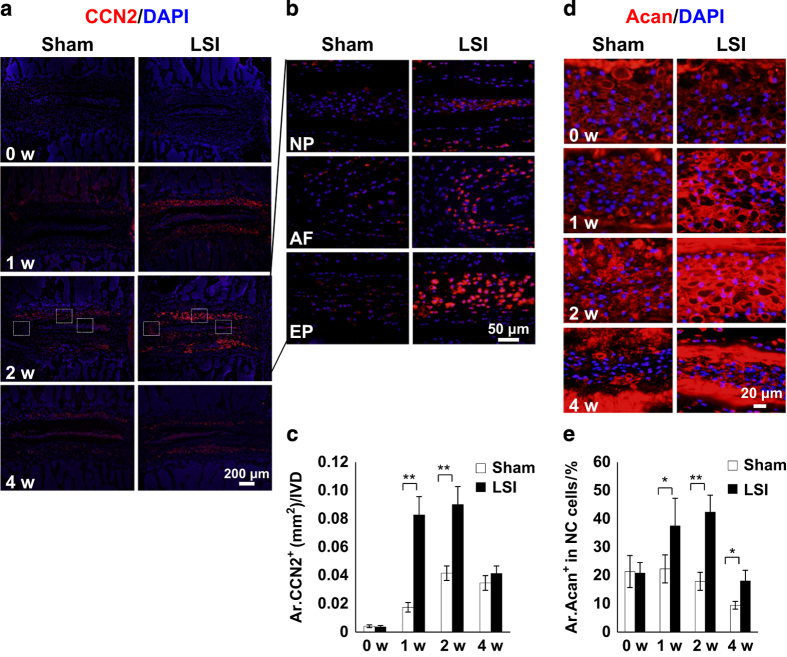
Mechanical instability stimulates extracellular aggrecan. (**a**) Immunofluorescence staining for CCN2 expression (red). DAPI stains nuclei blue. (**b**) Enlarged images of CCN2 expression in NP, AF and EP at 2 weeks post surgery. (**c**) Quantification of CCN2 expression in **a**. (**d**) Representative images of immunofluorescence staining for aggrecan (Acan, red). DAPI stains nuclei blue. (**e**) Quantitative analysis of Acan^+^ area in NC cells. *n=6* per group. Data are shown as mean±s.d. *P<0.05, **P<0.01 (two-sided Student’s *t* test).

The IVD height increased in mice from birth through 1 month, and then remained stable before decreasing around 4 months. The NC cells’ sphere-shaped vacuole observed at birth changed to a spindle-like shape by 4 months (Figure 1a and c). Extracellular aggrecan secretion was stimulated on day 3 after birth and accumulated around the NC cells to generate IVD extracellular space adjacent to endplates from day 7 (Figure 1b). To investigate that these changes were associated with mechanical stress, we employed a LSI mouse model by removing spinous processes and posterior supraspinous and interspinous ligaments (Figure 1d).^28,29^ The effects of mechanical stress on NC cells were analyzed by immunostaining of the IVD sections collected from 2-month-old LSI mice, in which the IVD space is peaked. The vacuole sizes of NC cells gradually decreased beginning at 2 weeks post surgery in LSI, whereas in the sham-operated mice NC size decreased at 4 weeks with Safranin O staining (Figure 1e). Significant IVD degeneration was observed in LSI mice relative to sham-operated mice, as shown by IVD score beginning as early as 1 week post surgery^1,38^ (Figure 1g). Mechanical stress has been shown to activate latent TGFβ.^20,21,39,40^ Immunostaining revealed that phosphorylated Smad2/3-positive cells (pSmad2/3^+^) in the NP were significantly increased 1, 2, and 4 weeks post surgery in the LSI mice relative to the sham controls (Figure 1f and h). Further, the levels of CCN2, a TGFβ downstream factor that upregulates the synthesis of matrix proteins in IVDs,^26,27,41–43^ were significantly increased 2 weeks post surgery in LSI mice and gradually decreased to sham control levels by 4 weeks, consistent with the increase of TGFβ activity (Figure 2a–c). Similarly, aggrecan expression, upregulated by CCN2,^26,44^ was also increased in LSI mice (Figure 2d and e). Taken together, the data reveal that increase of TGFβ activity in response to mechanical stress stimulates secretion of extracellular proteins such as aggrecan. Simultaneously, a reduction in intracellular vacuoles was observed with a transition of IVD space maintained by intracellular vacuoles in early postnatal life versus extracellular matrix in adulthood or with increased mechanical stress.

### Integrin α_v_β_6_ induces TGFβ activation in response to mechanical stress to regulate NC cell function

**Figure 3:**
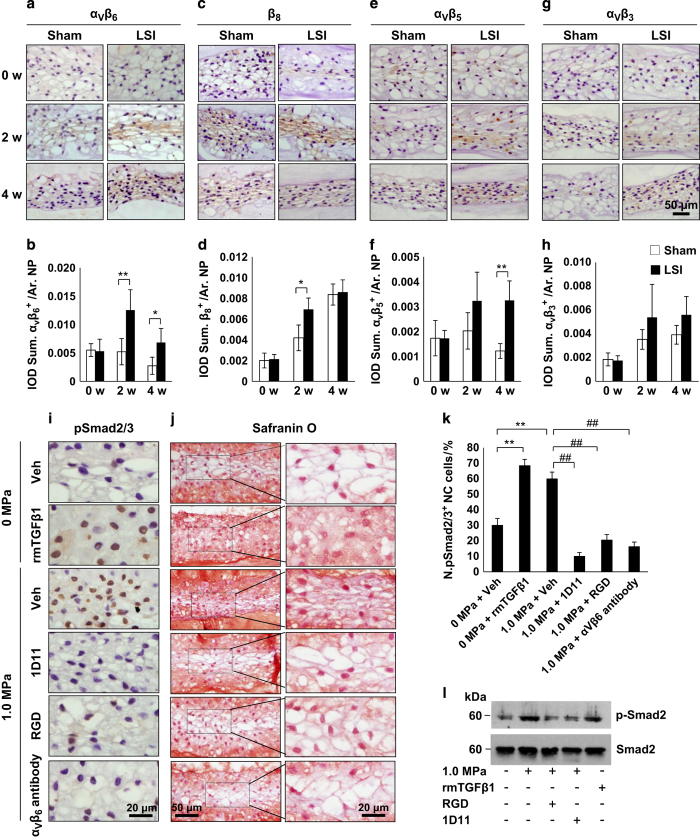
Integrin α_V_β_6_ induces TGFβ activation in response to mechanical stress. (**a**–**h**) Representative images and quantification of immunostaining of IVD sections with antibodies against (**a**, **b**) α_V_β_6_, (**c**, **d**) β8, (**e**, **f**) α_V_β_5_, and (**g**, **h**) α_V_β_3_ (brown) in LSI and sham mice. Hematoxylin stains nuclei purple. *n*=6 per group. Data are shown as mean±s.d. *P<0.05, **P<0.01 (two-sided Student’s *t* test). (**i**) Immunostaining for pSmad2/3 in the NC cells (brown). Hematoxylin stains nuclei purple. (**j**) Safranin O staining of IVD sections from an IVD *ex vivo* compression model with application of either Veh (rows 1 and 3), recombinant mouse TGFβ1 (row 2), RGD peptide, TGFβ or α_V_β_6_ neutralizing antibodies (bottom 3 rows). (**k**) Quantification of pSmad2/3^+^ cells in **i**. (**l**) Western blot analysis of pSmad2 and total Smad2 levels in the IVD. *n*=6 per group in histological examination. Representative image from three independent experiments were conducted for (**l**). Data are shown as mean±s.d. *P<0.05, **P<0.01 (ANOVA).

We then investigated the mechanism of mechanical stress-induced activation of latent TGFβ. The α_v_β integrins are one known mechanism that mediates cell-induced conformational change of TGFβ latent complex to release active TGFβ.^21–24,45,46^ The α_v_ integrin is the common α subunit for β integrins. Immunostaining of IVD sections revealed that the expression of one specific β integrin, α_v_β_6_, in the NP was significantly increased 2 and 4 weeks post surgery in LSI mice relative to sham control (Figure 3a and b). The pattern of elevation of α_v_β_6_ expression was similar to the increase of pSmad2/3-positive cells (Figure 1f and h), whereas the expression of β_8_, α_v_β_5_, and α_v_β_3_ did not correlate (Figure 3c–h).

**Figure 4:**
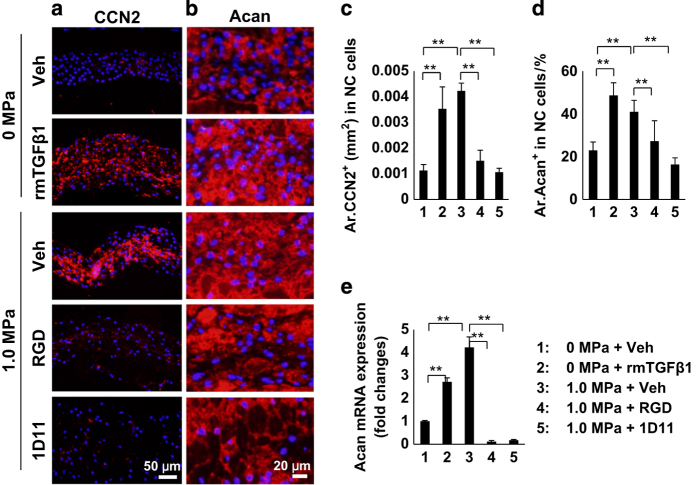
Mechanical stress regulates NC cell function. (**a**, **b**) Immunofluorescence staining of IVD sections for (**a**) CCN2 and (**b**) Acan in the NP (red). DAPI stains nuclei blue. (**c**, **d**) Quantification of CCN2 and Acan expression in **a** and **b**. (**e**) Expression of Acan mRNA in NP tissues by quantitative RT-PCR (qRT-PCR). *n*=6 per group in histological examination. Three independent experiments performed in triplicate were conducted for e. Data are shown as mean±s.d. *P<0.05, **P<0.01 (ANOVA).

To determine a causal relationship, active TGFβ, RGD peptide, neutralizing antibodies against TGFβ orα_v_β_6_, or vehicle were applied to an *ex vivo* IVD compression loading model.^37^ Immunostaining demonstrated that RGD peptide, antibodies against TGFβ (1D11), or α_v_β_6_ all inhibited stress-induced phosphorylation of Smad2/3 in NC cells (Figure 3i and k). The result was confirmed by western blot analysis (Figure 3l). Importantly, the morphology of NC cell vacuoles was altered in the active TGFβ and vehicle-compression treatment groups (Figure 3j, second and third row), whereas the sphere shape of vacuoles was maintained in the RGD, TGFβ (1D11), and α_v_β_6_ antibody groups (Figure 3j, bottom three rows) relative to the zero compression vehicle group (Figure 3j, top row). In contrast, the extracellular CCN2 and aggrecan were significantly stimulated in the active TGFβ group and compression group treated with vehicle (Figure 4a and b, rows 2 and 3; Figure 4c and d) but inhibited in the RGD and TGFβ antibody groups (Figure 4a and b, rows 4 and 5). Messenger RNA expression of aggrecan was similarly inhibited by RGD and TGFβ antibody (Figure 4e).

### Conditional knockout of TGFβ type II receptor prohibits functional transition of NC cells

**Figure 5:**
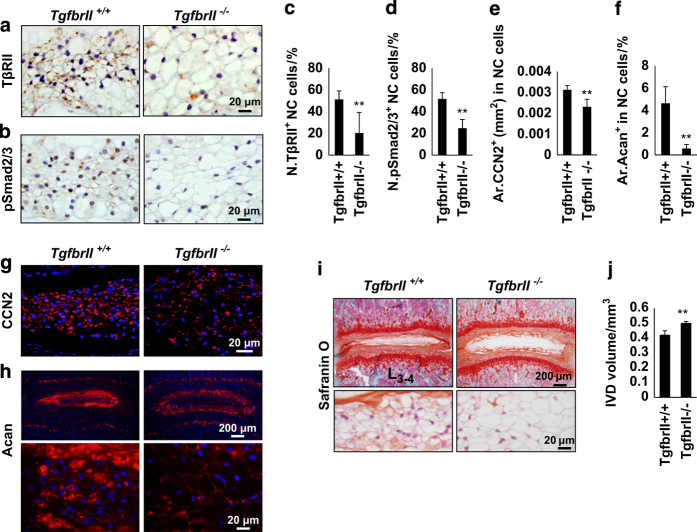
Conditional knockout of TGFβ type II receptor prohibits functional transition of NC cells. (**a**, **b**) Immunostaining of IVD sections from *TgfbrII*^*−/−*^ and their *TgfbrII*^*+/+*^ littermates with antibodies against TβRII and pSmad2/3 (brown). Hematoxylin stains nuclei purple. (**c**, **d**) Quantification of TβRII- and pSmad2/3-positive cells in **a** and **b**. (**g**, **h**) Immunofluorescence staining of IVD sections for CCN2 (**g**) and Acan (**h**) (red). DAPI stains nuclei blue. (**e**, **f**) Quantification of CCN2 and Acan in the NC cells in g and h. (**i**) Safranin O-fast green staining of L_3–4_ IVDs in *TgfbrII*^*−/−*^ mice and their *TgfbrII*^*+/+*^ littermates (4-week-old). (**j**) Quantitative analysis of IVD volumes from μCT scan. *n*=6 per group. Data are shown as mean±s.d. *P<0.05, **P<0.01 (two-sided Student’s *t* test).

To confirm the role of TGFβ in control of NC cell function *in vivo*, notochord-originated cell-specific *TgfbrII* knockout mice (*TgfbrII*^−/−^) were generated by crossing floxed *TgfbrII* mice with *Noto-Cre* mice. TβRII*-* and pSmad2/3-positive NC cells were almost undetectable in immunostaining of IVD sections of *TgfbrII*^−/−^ mice (Figure 5a–d), validating our mouse model. Immunostaining demonstrated that CCN2 and aggrecan were much lower in the NPs of *TgfbrII*^−/−^ mice relative to WT *TgfbrII*^+/+^ littermates (Figure 5e–h), further confirming CCN2 and aggrecan as downstream signaling targets. Safranin O staining of IVD sections showed that NC cells have much larger vacuoles with less proteoglycan in 4-week-old *TgfbrII*^−/−^ mice relative to their WT *TgfbrII*^+/+^ littermates (Figure 5i). Furthermore, the IVD volume was significantly increased in the *TgfbrII*^−/−^ mice in comparison to their WT *TgfbrII*^+/+^ littermates in μCT analysis (Figure 5j). These genetic data demonstrate that TGFβ signaling in NC cells affects vacuole size for IVD morphology.

### Conditional knockout of integrin α_v_ impedes postnatal functional transition of NC cells

**Figure 6:**
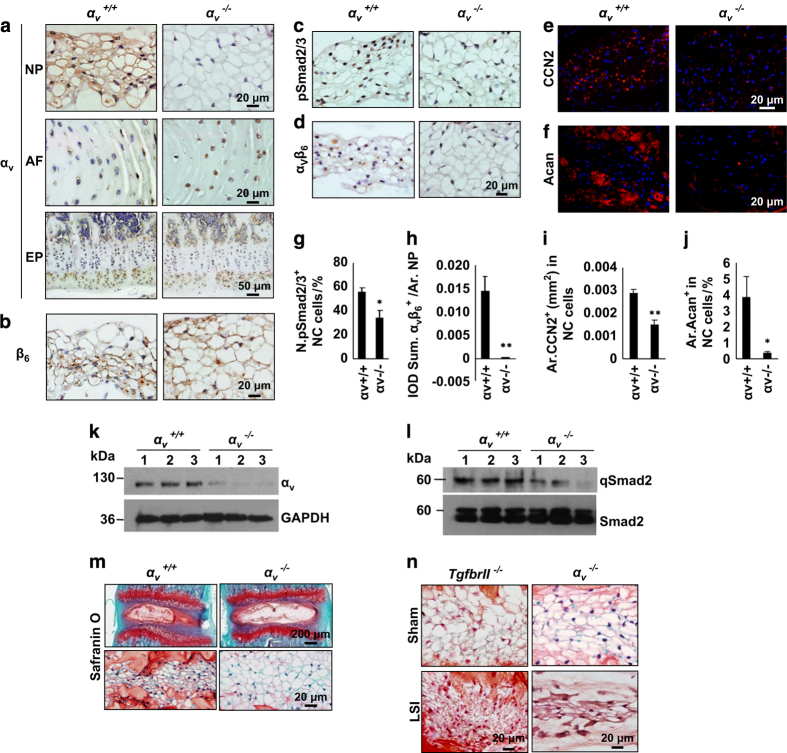
Conditional knockout of integrin α_v_ impedes functional transition of NC cells. (**a**, **b**) Immunostaining of IVD sections from α_v_^−/−^ mice and their wild-type α_v_^+/+^ littermates (4-week-old) with antibodies against α_v_ (**a**) and β_6_ (**b**) (brown). Hematoxylin stains nuclei purple. (**c**, **d**) Immunostaining for pSmad2/3 (**c**) and α_V_β_6_ (**d**) in NC cells. (**e**, **f**) Immunostaining for CCN2 (**e**) and Acan (**f**) in NC cells. (**g**, **h**) Quantification of pSmad2/3 (**c**) and α_V_β_6_ (**d**) expression in NC cells. (**i**, **j**) Quantification of CCN2 (**i**) and α_V_β_6_ (**j**) expression in NC cells. (**k**) Western blot analysis of α_v_ level in NC cells. (**l**) Western blot analysis of pSmad2 and Smad2 levels in NC cells. (**m**) Safranin O-fast green staining of L_3–4_ IVDs showing enlarged vacuoles with less proteoglycans (red orange) in α_v_^−/−^ mice relative to their α_v_^+/+^ littermates (4-week-old). (**n**) Safranin O staining images of the IVDs from sham-operated or LSI *TgfbrII*^*−/−*^ mice and α_v_^−/−^ mice (8-week-old) at 2 weeks post surgery. *n*=3 per group. Data are shown as mean±s.d. *P<0.05, **P<0.01 (two-sided Student’s *t* test).

We then examined the role of integrin α_v_ in activation of TGFβ in notochord cells as α_v_ chain is the core to mediate the activation of TGFβ. Floxed α_*v*_ mice were crossed with *Noto-Cre* mice to generate α_*v*_ knockout mice (α_*v*_^−/−^). Immunostaining of IVD sections validated that α_v_ expression was diminished specifically in NC cells, whereas the expression remained unchanged in AF and EP cells in α_*v*_^−/−^mice relative to their WT α_*v*_^+/+^ littermates (Figure 6a). Integrin β_6_ expression was unaffected in the α_*v*_^−/−^ NC cells (Figure 6b). The reduction of α_v_ in the α_*v*_^−/−^ NC cells was further confirmed by western blot analysis (Figure 6k). As expected, α_v_β_6_- and pSmad2/3-positive NC cells were significantly reduced in α_*v*_^−/−^mice relative to WT α_*v*_^+/+^ littermates (Figure 6c, d, g and h). The contents of CCN2 and aggrecan were also much lower in the NPs of α_*v*_^−/−^ mice relative to WT α_*v*_^+/+^ littermates (Figure 6e, f, i and j). Similar to *TgfbrII*^−/−^mice, NC cells had much larger vacuoles with less proteoglycan in the α_*v*_^−/−^ mice relative to their WT littermates (Figure 6m). The α_*v*_^−/−^ and *TgfbrII*^−/−^ mice demonstrate that disruption of TGFβ signaling can delay NC morphologic transition. Therefore, to evaluate whether the morphologic change of vacuoles was sufficient to prevent mechanically induced IVD degeneration, we further subjected α_*v*_^−/−^ and *TgfbrII*^−/−^ mice to LSI or sham surgery (Figure 1d). Safranin O staining showed that IVD was significantly degenerated in both LSI α_*v*_^−/−^ and *TgfbrII*^−/−^ mice relative their sham-operated controls (Figure 6n), and demonstrated that complete loss of NC cell TGFβ signaling can also be as detrimental as excessive TGFβ signaling.

### Inhibition of excess activation of TGFβ attenuates IVD degeneration

**Figure 7:**
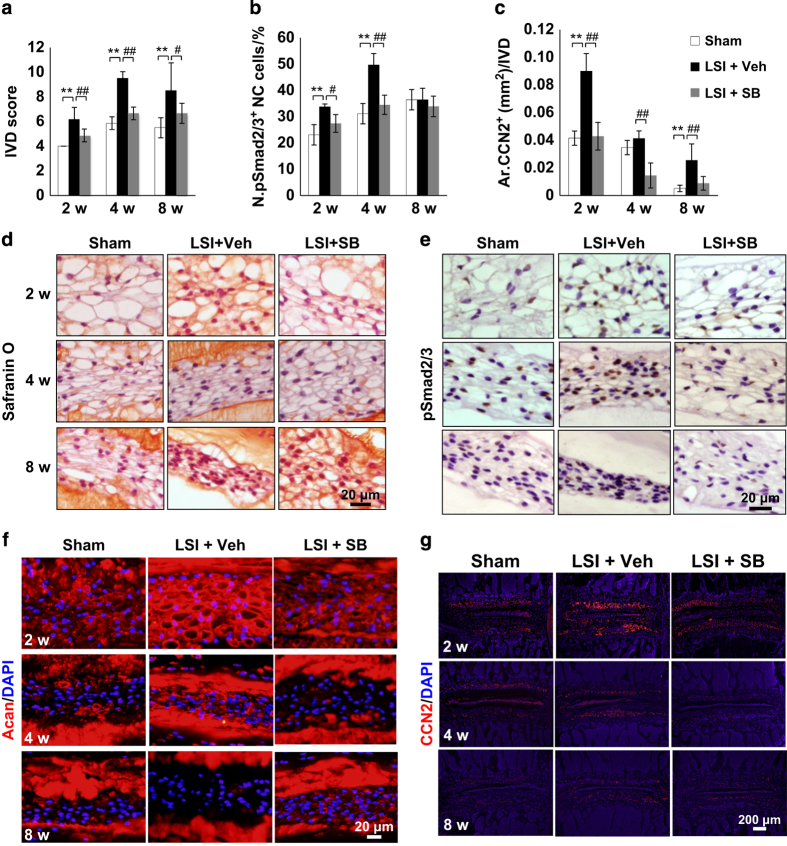
Inhibition of excess active TGFβ attenuates IVD degeneration in LSI mice. (**a**) IVD score in sham, LSI mice with injection of either Veh or TβRI inhibitor (SB, 1.0 mg·kg^−1^). (**b**, **e**) Immunostaining and quantification of pSmad2/3 in the NP (brown). Hematoxylin stains nuclei purple. (**c**) Quantification of CCN2 areas in **g**. (**d**) Safranin O staining of L_4–5_IVD section. (**f**, **g**) Immunofluorescence staining of IVD sections for Acan (**f**) and CCN2 (**g**) (red). DAPI stains nuclei blue. *n*=6 per group. Data are shown as mean±s.d. *P<0.05, **P<0.01, ^#^*P*<0.05, ^##^*P*<0.01 (ANOVA).

We examined whether inhibition of excessive active TGFβ is beneficial for IVD degeneration. TGFβ type I receptor (TβRI) inhibitor (1 mg·kg^−1^) was intraperitoneally injected in the LSI mice post surgery. Degeneration of IVD was attenuated as assessed by IVD score (Figure 7a). The vacuolar morphology was preserved with the TβRI inhibitor treatment relative to sham-operated versus LSI-operated with vehicle treatment (Figure 7d). As expected, pSmad2/3^+^ cells were reduced by TβRI inhibitor (Figure 7b and e). Expression of aggrecan and CCN2 was maintained at similar levels of sham-operated mice as opposed to the elevation of both seen in the LSI-operated vehicle-treated group (Figure 7c, f and g).

**Figure 8:**
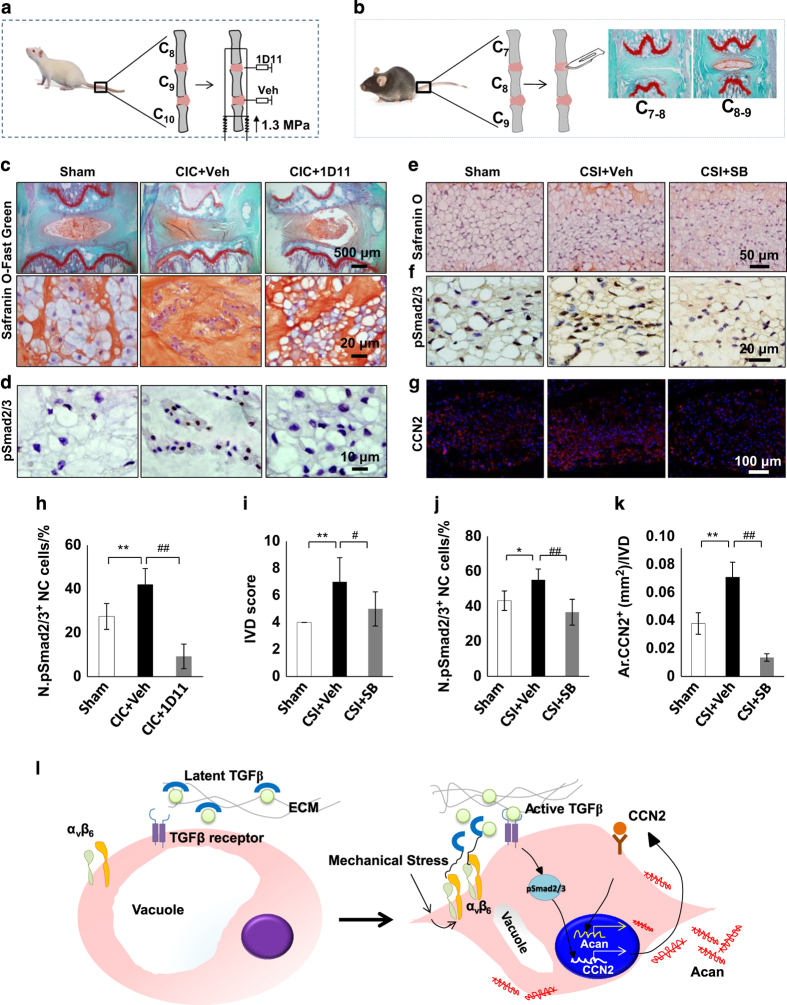
Inhibition of excess active TGFβ attenuates IVD degeneration in CIC rats and CSI mice. (**a**, **c**, **d**, **h**) Local administration of TGFβ neutralizing antibody (1D11, 0.7 μg) into the NP of rat caudal IVD compression model (CIC, 12-week-old) prevented IVD degeneration. (**a**) Rat CIC model. Rats were attached with the loading device. The axial stress loaded from the distal side produced a compressive pressure of 1.3 MPa on C_8–9_ and C_9–10_ IVDs. The C_8–9_ of loaded IVDs were injected with an alginate bead containing 1D11 while the C_9–10_ IVDs with that containing vehicle. (**c**) Safranin O staining of Caudal 8th-9th (C_8–9_) IVD with 1D11 or vehicle treatment. Immunostaining and quantification of pSmad2/3^+^ cells (**d**, **h**) in the NP of CIC rat (brown). Hematoxylin stains nuclei purple. *n*=6 per group. (**b**, **e** **g**, **i** **k**) Caudal SI(CSI) mouse model was induced by full-depth annular stab and NP removal of the C_7–8_ IVD. (**b**) CSI mouse model. The instability of caudal spine was induced by full-depth annular stab and NP removal of the C_7–8_ IVD. The adjacent C_8–9_ IVDs were chosen for observation. The adjacent C_8–9_ IVDs were chosen for observation. (**e**) Safranin O staining of IVD section. Immunostaining of C_8–9_IVD sections with antibodies against (**f**) pSmad2/3 and (**g**) CCN2; (**i**) IVD degeneration was evaluated by IVD score at 4 weeks post surgery. Quantification of (**j**) pSmad2/3^+^ cells and (**k**) CCN2^+^ expression in **f** and **g**. *n*=6 per group. Data are shown as mean±s.d. *P<0.05, **P<0.01, ^#^*P*<0.05, ^##^*P*<0.01 (ANOVA). (**l**) Model showing mechanosignaling activation of TGFβ controls IVD homeostasis.

In parallel, we administrated the TGFβ-neutralizing antibody directly into the IVD in a rat caudal IVD compression model.^31–34^ (Figure 8a) Neutralizing active TGFβ in the IVD prevented IVD degeneration as defined by preservation of the NC cells morphology under static compression relative to vehicle treatment (Figure 8c). The expression pSmad2/3^+^ in the NC cells was reduced with TGFβ antibody treatment compared to vehicle under static compression (Figure 8d and h). Similar results were obtained using a caudal spine instability mouse model by intraperitoneal injection of TβRI inhibitor^30,47,48^ (Figure 8b), in which IVD degeneration was effectively prevented (Figure 8e–g and 8i–k ).

## Discussion

The initiation of DDD by NP dysfunction is a hot topic in the spinal disease field. NP cells originate from the notochord. During development, NC cells regulate the spinal morphogenesis by control of intracellular vacuoles evenly distributed in the NP to generate intervertebral space.^6,10,49,50^ After birth, IVDs gradually change their content with a reduction of intracellular vacuoles of NC cells corresponding to increase extracellular proteoglycans.^15^ Loss of vacuolar NC cells and their morphologic and functional transition are thought as early degenerative changes of IVD.^12^ However, the mechanism of NC cells transition is not well understood. In this study, we revealed that integrin α_v_β_6_-induced activation of TGFβ controls the functional transition of NC cells in IVDs in multiple rodent models. Active TGFβ reduced intracellular vacuoles of NC cells, but increased extracellular proteoglycans, thereby shifting the burden of mechanical loads from the cells to the extracellular environment.

Mechanical loads are one of the most important etiologies of DDD.^50^ In normal physiological situations, appropriate mechanical loading produced by body weight and muscle force is necessary to activate TGFβ in the NP to modulate anabolic activity of NC cells and maintain IVD homeostasis, similar to its role in homeostasis of the bone and cartilage.^40,51–53^ However, in pathological conditions, as we found in the present study, compression stresses activate excess TGFβ and result in accelerated functional transition of NC cells, leading to pathologic changes of IVD.

Central to this mechanosignal transduction is the activation of TGFβ by integrin α_v_β_6_. Several of integrin α and β chains have been found in IVD involving in cell–matrix interaction.^25,54^ Integrin α_5_β_1_ has recently been shown to be associated with mechanotransduction in IVD cells.^55^ Mechanical stress has been shown to induce cell cytoskeleton changes, leading to integrin α_v_β_6_ binding to the latent TGFβ complex for its conformational change to allow active TGFβ to bind to its receptor.^45^ Similarly, we found it is integrin α_v_β_6_ that correlates to active TGFβ signaling pathway and regulates NC cells function by mechanical stimili. TGFβ signaling activates transcription and secretion of CCN2, which in turn binds to CCN2 receptor to stimulate a cascade of changes in extracellular proteoglycan expression.^56^ Aggrecan, the major proteoglycan in IVD, exerts osmotic pressure to resist compressive loads.^57^ However, aggrecan also affects the osmotic pressure in the IVD. Besides shifting water from outside the IVD, our study suggests that aggrecan likely also results in a shifting of water from intracellular to extracelluar matrix and leads to NC cells morphologic transition by dehydration.

TGFβ is recognized as an anabolic factor in IVD and has been found to prevent DDD.^58^ However, high levels of TGFβ1 have also been observed in the IVDs from DDD patients.^39,40,59–63^ One group has previously shown that inhibition of TGFβ can prevent DDD, which was attributed to a change in pSmad1/5/8 to pSmad 2/3 downstream signaling.^64^ The various effects of TGFβ are likely related to the concentration of TGFβ. Our results demonstrated that pathologic mechanical loading on the spine drove aberrant overactivation of TGFβ, resulting in DDD. Complete inhibition of TGFβ signaling in knockout of TβRII or α_v_ mice also caused IVD degeneration by mechanical stimuli. Thus, TGFβ regulates IVD cell function and homeostasis, whereas either high or low concentrations of TGFβ led to DDD development.

DDD is a polygenic disease exacerbated by many different factors.^2,16,50,64–68^ In this study, we found that under mechanical stress, αvβ6 integrin is activated and can release TGFβ from its latent form. Activation of TGFβ signaling pathway in multiple mouse models increased the expression of CCN2, which then upregulated aggrecan. High levels of aggrecan likely increase the osmotic pressure of the extracellular environment to result in a shift in vacuole liquid volume, resulting in an apparent change in NC cell morphology, from vacuole-like to fibroblast-like cells (Figure 8l). Reduction of aberrant TGFβ overactivation in the IVDs through modulation of mechanical stress, or inhibition of either α_v_β_6_ or TGFβ signaling, could have therapeutic potential for DDD.
